# A Natural Inhibitor, 1′*S*-1′-Acetoxychavicol Acetate, Against Testosterone-Induced Alopecia via NADPH Oxidase Regulation

**DOI:** 10.3390/molecules30102246

**Published:** 2025-05-21

**Authors:** Kkotnara Park, Isoo Youn, Jung Min Suh, Min Hye Choi, Da-Woon Bae, Soo-Bong Park, Mi Hee Kwack, Sun-Shin Cha, Dae Sik Jang, Young Kwan Sung, Yun Soo Bae, Eun Kyoung Seo

**Affiliations:** 1 Department of Life Sciences, Ewha Womans University, Seoul 03760, Republic of Korea; 2 College of Pharmacy, Ewha Womans University, Seoul 03760, Republic of Korea; 3 Department of Chemistry and Nanoscience, Ewha Womans University, Seoul 03760, Republic of Korea; 4 Department of Immunology, School of Medicine, Kyungpook National University, Daegu 41566, Republic of Korea; 5 Department of Pharmaceutical Science, College of Pharmacy, Kyung Hee University, Seoul 02447, Republic of Korea

**Keywords:** reactive oxygen species, NADPH oxidase, Nox inhibitor, 1′*S*-1′-acetoxychavicol acetate, androgenetic alopecia

## Abstract

Androgenetic alopecia is associated with testosterone-mediated anagen-to-catagen transition and matrix keratinocyte apoptosis in hair follicle cells. Activation of Nox isozymes is involved in testosterone-mediated keratinocyte apoptosis, leading to androgenetic alopecia. This indicates that Nox isozymes can serve as therapeutic targets for androgenetic alopecia. The isolated compounds from natural products were screened to evaluate their ROS-inhibition efficacy and it was found that 1′*S*-1′-acetoxychavicol acetate (ACA, **26**), a natural compound isolated from *Alpinia galanga* (L.) Willd. (Zingiberaceae), exhibits inhibitory activity on Nox isozymes. Nox inhibition by ACA suppressed testosterone-dependent H_2_O_2_ generation and cell death in keratinocytes. Incubation with ACA in human hair follicle organ culture mitigated testosterone-dependent suppression of hair growth. We validated that ACA regulates androgenetic alopecia in a mouse model. Local application of ACA on the dorsal skin in an androgenetic alopecia model of C57BL/6 mice significantly suppressed testosterone-induced hair loss in a dose-dependent manner. Moreover, hair follicle length in ACA-treated mice was enhanced compared to that in control mice. These findings provide a molecular mechanism in which ACA inhibits Nox activity in hair follicle cells, indicating its potential as an effective treatment of AGA.

## 1. Introduction

Hair growth is composed of four phases: anagen (growth), catagen (regression), telogen (rest), and exogen (shedding) [1]. Each hair follicle undergoes an independent cycle with ten to thirty cycles in its lifetime. Hair follicles are composed of cell types, including dermal papilla (DP), bulge, matrix, and root sheath cells. DP cells derived from mesenchymal stem cells are located at the bases of hair follicles and regulate hair growth by interacting with hair follicle epithelial cells. Matrix cells can differentiate into various cell lineages of the hair shaft and the inner root sheath, whereas the outer root sheath (ORS) is derived from epithelial progenitor cells [2,3]. Testosterone can regulate the cycle of hair follicles by stimulating the anagen-to-catagen transition, promoting hair loss. Typical characteristics of catagen and telogen phases are apoptosis of ORS and regression of DP, and ORS cell death is mediated by testosterone-mediated reactive oxygen species (ROS) generation.

Androgenetic alopecia (AGA), also known as hair loss, commonly affects both men and women and is regulated by several factors, including hereditary traits, testosterone-related action, hair follicle miniaturization, and shortened anagen phase [4]. Among these factors, the genomic action of testosterone within the DP is a well-known pathological mechanism of AGA. To be specific, type Ⅱ 5*α*-reductase converts testosterone into 5α-dihydrotestosterone (DHT), and DHT can bind to cytoplasmic androgen receptor (AR) to pass through the nucleus. The DHT-AR complex can stimulate the expression of various factors such as vascular endothelial growth factor (VEGF), transforming growth factor β1 (TGF-*β*1), and Dickkopf-related protein 1 (DKK-1) [5,6,7]. Testosterone-dependent regulation of these factors is involved in premature anagen termination and early catagen induction in AGA.

In contrast to the genomic pathway of testosterone, several lines of evidence have indicated that G protein-coupled receptor family C group 6 member A (GPRC6A) can serve as a receptor for testosterone on the plasma membrane. GPRC6A has been identified as a receptor for *L*-amino acids, calcium, and osteocalcin [8,9]. The authors previously reported that testosterone can stimulate GPRC6A and activate a cascade of Gq-PLCβ-IP_3_-Ca^2+^ in epidermal keratinocytes [10]. On the other hand, nicotinamide adenine dinucleotide phosphate (NADPH) oxidase (Nox) is a multicomponent enzyme complex that produces ROS and seven homologues (Nox1, Nox2, Nox3, Nox4, Nox5, Duox1, and Duox2) have been identified. Duox1 is a predominant isozyme in keratinocytes and testosterone-induced intracellular calcium mobilization through GPRC6A can directly activate Duox1, resulting in the generation of H_2_O_2_ and keratinocyte apoptosis. These explanations suggest a molecular mechanism between non-genomic testosterone action and cellular redox status related to AGA.

Several lines of evidence indicate that oxidative stress within cells results from an imbalance between reactive oxygen species (ROS) production and elimination [11,12]. This imbalance is primarily caused by the overexpression of Nox isozymes, which promotes ROS generation, along with relatively impaired antioxidant systems responsible for ROS scavenging. Although many studies have focused on developing antioxidants to regulate intracellular oxidative stress, their effects on hair loss have been limited [13].

In the case of male pattern hair loss, ROS production through the testosterone-GPRC6A-Nox cascade promotes apoptosis of hair follicle cells [14]. If a natural compound that inhibits Nox activity can prevent this follicle cell apoptosis, it could pave the way for the development of a novel therapy for hair loss. Therefore, we screened a library of natural compounds using *drosophila* membrane expressing human Nox isozymes and then identified 1′*S*-1′-acetoxychavicol acetate (ACA, **26**) as having Nox inhibitory activity. The present study found that ACA could suppress testosterone-mediated ROS generation and cell death. Moreover, the addition of ACA into human hair follicle organ culture ameliorated testosterone-mediated hair growth inhibition. We provide a molecular mechanism by which ACA may be effective for treating AGA through Nox inhibition.

## 2. Results

### 2.1. Identification of ACA as an Inhibitor of Nox from a Library of Natural Compounds

**Figure 1:**
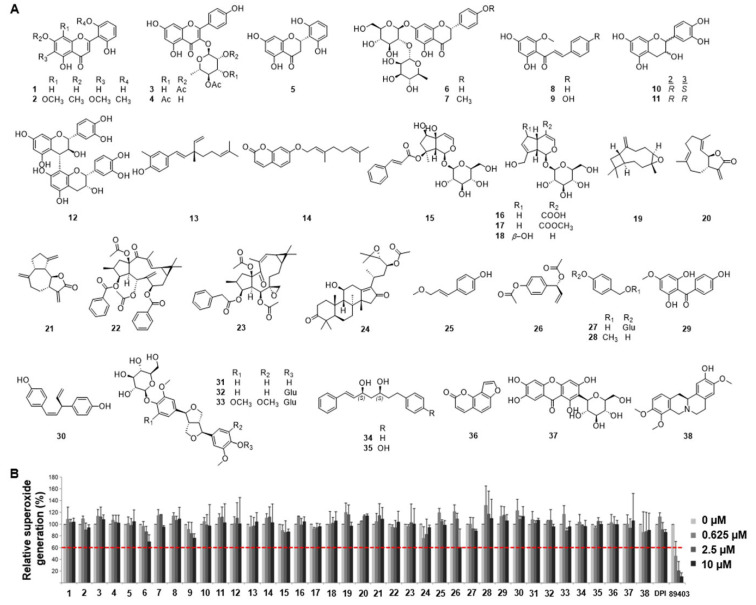
Reactive oxygen species (ROS) inhibition screening of thirty-eight compounds. (**A**) Structures of the compounds (**1–38**) isolated from plant materials. (**B**) Inhibition of ROS generation by the compounds. The red dotted line indicates the point at which 60 % of ROS generation occurs. Diphenyleneiodonium (DPI) and Ewha-89403 (89403) are positive controls for Nox inhibitors. 5,7,2′,6′-tetrahydroxyflavone (**1**); skullcapflavone Ⅱ (**2**); kaempferol 3-*O*-(2,4-*O*-diacetyl-*α*-L-rhamnopyranoside) (**3**); kaempferol 3-*O*-(3,4-*O*-diacetyl-*α*-L-rhamnopyranoside) (**4**); (2*S*)-5,7,2′,6′-tetrahydroxyflavanone (**5**); naringin (**6**); poncirin (**7**); cardamonin (**8**); helichrysetin (**9**); (+)-catechin (**10**); (-)-epicatechin (**11**); procyanidin B_4_ (**12**); (*S*)-bakuchiol (**13**); auraptene (**14**); harpagoside (**15**); geniposidic acid (**16**); geniposide (**17**); aucubin (**18**); *β*-caryophyllene oxide (**19**); costunolide (**20**); dehydrocostus lactone (**21**); euphorbia factor L_2_ (**22**); euphorbia factor L_1_ (**23**); alisol C 23-acetate (**24**); (*E*)-*p*-coumaryl alcohol methyl ether (**25**); 1′*S*-1′-acetoxychavicol acetate (**26**); gastrodin (**27**); 4-methoxybenzyl alcohol (**28**); 2,6,4′-trihydroxy-4-methoxybenzophenone (**29**); nyasol (**30**); pinoresinol 4-*O*-*β*-D-glucoside (**31**); pinoresinol diglucoside (**32**); liriodendrin (**33**); (1*E*,3*S*,5*S*)-1,7-diphenyl-1-heptene-3,5-diol (**34**); (3*S*,5*S*)-alpinikatin (**35**); isopsoralen (**36**); mangiferin (**37**); (-)-isocorypalmine (**38**); DPI; 89403.

Several lines of evidence indicate that testosterone-dependent activation of the GPRC6A cascade results in activation of Duox and intracellular ROS generation [14]. These events can cause apoptosis of ORS cells and primary epidermal keratinocytes, leading to AGA. Based on the hypothesis that testosterone-dependent apoptosis of ORS cells is mediated by Nox-induced ROS generation, a Nox inhibitor screening assay was performed by recording luminescence intensity derived from lucigenin oxidation. Mouse kidney membranes expressing Nox1, Nox2, Nox4, or Duox1 isozymes were incubated with natural compounds (1-38, Figure 1A), and diphenyleneiodonium (DPI) and Ewha-89403 (89403) were used as controls. ROS generation was then measured by lucigenin chemiluminescence (Figure 1B). Among the tested compounds, ACA (26) isolated from *Alpinia galanga* showed potent activity, with 60% inhibition of ROS generation at 10 μM.

**Figure 2:**
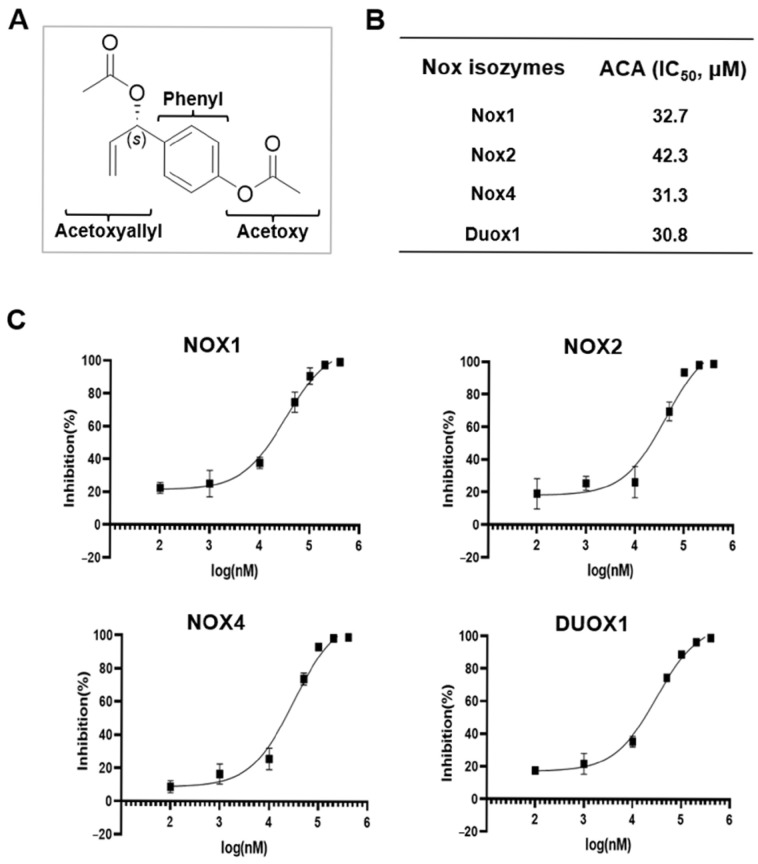
Identification of 1′*S*-1′-acetoxychavicol acetate (ACA) as a Nox inhibitor. (**A**) Structural information of ACA. (**B**) IC_50_ values of ACA against Nox1, Nox2, Nox4, and Duox1. Lucigenin chemiluminescence assay was performed with ACA in *Drosophila* membrane expressing hNox1, hNox2, hNox4, and hDuox1. (**C**) Dose-inhibition curves of ACA to Nox1, Nox2, Nox4, and Duox1.

ACA was first identified from *Alpinia galanga* and *Alpinia conchigera,* typically used as traditional spices in cooking and medicines. The structural characteristics of ACA are shown in Figure 2A. To investigate the inhibitory activity of ACA against each Nox isozyme, purified *Drosophila* membranes expressing human Nox1, Nox2, Nox4, and Duox1 were used to monitor ROS production by determining the luminescence of lucigenin in the absence or presence of ACA. The IC_50_ values of ACA were 32.7 μM for hNox1, 42.3 μM for hNox2, 31.3 μM for hNox4, and 30.8 μM for hDuox1 membrane (Figure 2B). Therefore, ACA showed pan-Nox inhibitory activities against Nox1, Nox2, Nox4, and Duox1 isozymes. Dose-inhibition (%) curves of ACA against Nox1, Nox2, Nox4, and Duox1 are shown in Figure 2C.

### 2.2. Molecular Modeling of ACA with Nox5

**Figure 3:**
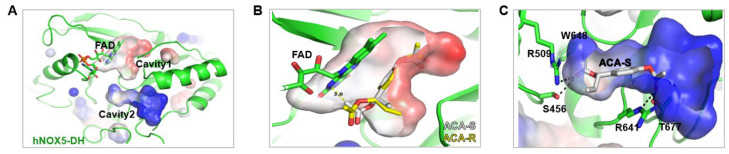
In silico docking structure of ACA with Nox5. (**A**) The modeled hNOX5-DH exhibits two distinct cavities denoted as Cavity 1 and Cavity 2. (**B**) In silico docking model of (*S*)- and (*R*)-ACA with Cavity 1 of Nox5. (**C**) In silico docking model of (*S*)-ACA in Cavity 2 of Nox5.

As the structures of Nox enzymes are very similar, the crystal structure of Nox5 originating from *Cylindrospermum stagnale* (*cs*Nox5) was used as a template and the structure of hNox5-DH was modeled with the Swiss-Model server. The dehydrogenase domain (DH) of *cs*Nox5 consisted of flavin adenine nucleotide (FAD)-binding and NADPH-binding lobes [15]. The modeled hNox5-DH exhibited two distinct cavities: Cavity 1 and Cavity 2 (Figure 3A). Cavity 1 is located between two lobes and is likely to serve as the binding site for the nicotinamide moiety of NADPH, stacking against the isoalloxazine moiety of FAD [15]. The positively charged Cavity 2 is postulated to accommodate the phosphate group of NADPH. As shown in Figure 3B, the in silico docking model revealed that (*S*)-ACA lacked appropriate conformations for Cavity 1, although the phenyl ring in (*S*)-ACA was anticipated to stack against the isoalloxazine moiety of FAD. When (*S*)-ACA was superposed onto the in silico docking result of (*R*)-ACA, whose phenyl ring stacked against the isoalloxazine moiety of FAD, the acetoxyallyl group of the *S* form sterically clashed with FAD due to their different stereochemistries. Instead, it occupied Cavity 2 with the lowest free energy level (−8.2 kcal/mol), as demonstrated in Figure 3C. According to the in silico docking model of (*S*)-ACA in Cavity 2, the methyl moiety of the acetoxyallyl group interacted with the indole ring of Trp648 and the aliphatic chain of Arg509. Moreover, the carbonyl oxygen of the acetoxyallyl group formed two hydrogen bonds, one with the guanidino group of Arg509 and the other with the hydroxyl group of Ser456. The carbonyl oxygen of the acetoxy group engaged in hydrogen bonding interactions with the guanidino group of Arg641 and the hydroxyl group of Thr677. Additionally, the phenyl ring of (*S*)-ACA is likely to establish an H-π interaction with the aliphatic chain of Arg641. In conclusion, these findings suggest that (*S*)-ACA exhibits inhibitory activity against hNox5 by disrupting the binding of the phosphate group of NADPH within Cavity 2 of hNox5-DH.

### 2.3. ACA Suppresses Testosterone-Mediated ROS Generation in Skin Keratinocytes

**Figure 4:**
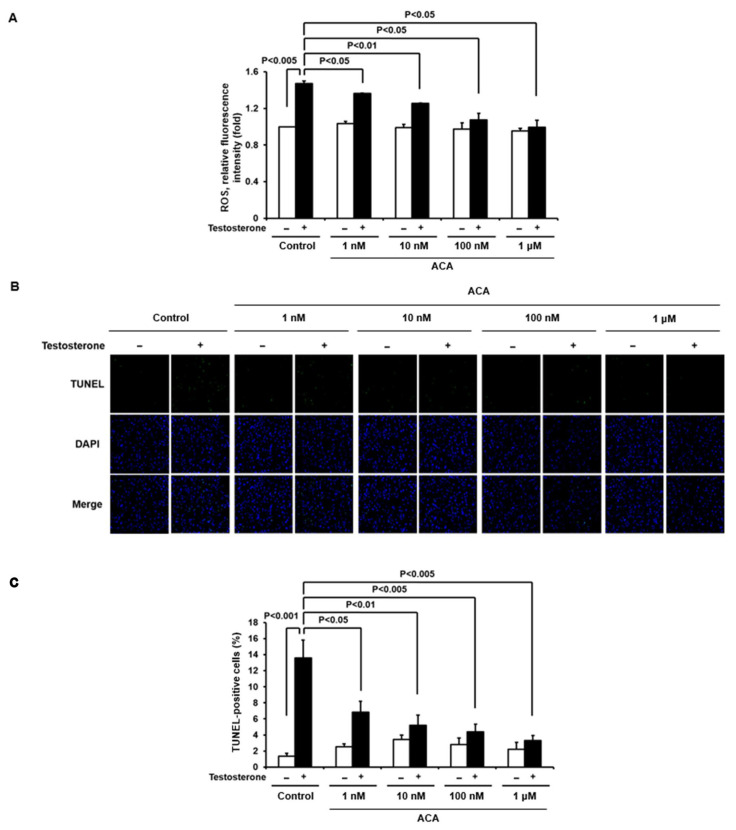
ACA inhibits testosteronemediated ROS production and apoptosis in keratinocytes. (**A**) Inhibitory activity of ACA in the testosterone-mediated ROS generation. Stimulation of Ker-CT cells with testosterone increased ROS generation, and then inhibitory activity of ACA in Ker-CT cells was determined using DCF-DA fluorescence intensity. Data are shown as mean ± SEM. (**B**) Apoptotic cells determined by TdT-UDP nick end labeling (TUNEL) assay. To investigate inhibitory activity of ACA in testosterone-induced apoptosis of skin keratinocytes, Ker-CT cells were incubated with or without 200 nM testosterone and ACA for 24 h. These cells were fixed and stained with TUNEL assay for 1 h at 37 °C. TUNEL-positive cells were visualized by fluorescence microscopy to identify and quantify apoptotic cells. DAPI staining for nuclei is blue fluorescence and TUNEL staining for apoptotic cells is green fluorescence. The percentage of apoptotic cells in eight random high-power fields was determined. (**C**) Quantitative analysis of TUNEL staining. Data are shown as mean ± SEM.

To evaluate whether ACA could suppress testosterone-mediated ROS generation in the cell line, intracellular levels of ROS in immortalized human keratinocytes (Ker-CT) were evaluated by measuring the fluorescence of DCF-DA with a confocal microscope (Figure 4A). Stimulation of Ker-CT cells with testosterone increased ROS generation. Pre-incubation with ACA at different doses inhibited the generation of ROS in Ker-CT cells in a dose-dependent manner in response to testosterone. Based on the inhibitory pattern of ACA on ROS generation, the EC_50_ value of ACA was 10.2 nM.

### 2.4. ACA Reduces Testosterone-Induced Apoptosis in Skin Keratinocytes

Previously, the authors reported that testosterone-dependent H_2_O_2_ generation is involved in the apoptosis of keratinocytes. To investigate whether ACA could inhibit testosterone-induced apoptosis, we used a TUNEL assay in hTERT-immortalized keratinocyte (Ker-CT) cells. Stimulation of Ker-CT cells with testosterone significantly increased TUNEL labeling compared to unstimulated cells, indicating that testosterone-dependent ROS generation induced apoptosis. Pretreatment of Ker-CT cells with the indicated concentration of ACA significantly suppressed testosterone-induced apoptosis (Figure 4B). The pattern of ACA inhibitory activity in testosterone-induced keratinocyte apoptosis was dose-dependent and the EC_50_ value of ACA was 5.8 nM (Figure 4C).

### 2.5. Function of ACA in Human Hair Follicle Organ Culture

**Figure 5:**
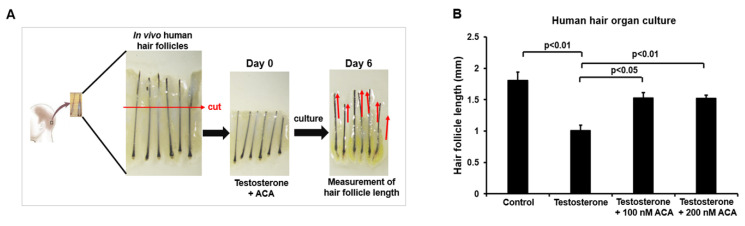
ACA attenuates testosterone-induced regression of hair growth in human hair follicle organ culture. (**A**) Schematic description of human hair follicle organ culture. (**B**) Hair growth suppression by ACA. Human non-balding hair follicles were cultured with testosterone and ACA for 6 days. Each group contained nine independent hair follicles. Red arrow indicate the direction of hair growth. Experiments were repeated three times. Data are shown as mean ± SEM.

To investigate the function of ACA in human hair follicle organ culture, hair follicles from male non-balding occipital scalps were cultured with testosterone in the absence or presence of ACA for 6 days (Figure 5A). Testosterone-treated human hair follicles showed suppression of hair growth. However, treatment with ACA mitigated this testosterone-dependent growth suppression (Figure 5B).

### 2.6. ACA Regulates Testosterone-Induced Hair Loss

**Figure 6:**
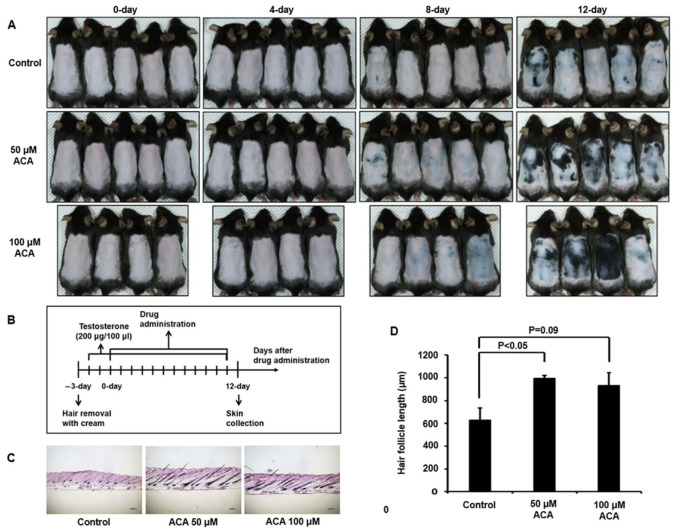
Function of ACA in hair growth from an androgenetic alopecia mouse model. (**A**) Images of dorsal skin of mice treated with ACA for the indicated periods. Dorsal skins of 8-week-old C57BL/6 mice were daily treated with topical application of testosterone (200 μg) for 3 days and then co-treated with ACA (50 μM or 100 μM) for 12 days. (**B**) Scheme of the experiment. (**C**) Dorsal skin tissues of androgenetic alopecia mice treated with ACA for 12 days after staining with H&E. Scale bar = 200 μm. (**D**) Hair follicle lengths of ACA-treated groups or control groups for 12 days. Data shown are mean (±SEM) of results from 4 to 5 independent sample preparations.

To validate its therapeutic effect on hair loss, ACA was applied to the dorsal skin of a testosterone-induced hair loss mouse. The telogen phase was observed in the dorsal skin of C57BL/6 male mice ranging from 7 weeks old to 12 weeks old. C57BL/6 male mice (8 weeks old) were administered testosterone for 3 days after removing dorsal hair. Two concentrations of ACA (50 µM and 100 µM) were administered to the dorsal skin of each testosterone-treated mouse for 12 days. As shown in Figure 6A, hair follicles were observed to grow in the ACA-treated group from 6–8 days, changing the skin from pink to gray. Finally, the skin turned black (indicating hair regrowth) on day 12. The scheme of hair growth measurement from an AGA mouse model is shown in Figure 6B. Later, skin tissues were collected and stained with H&E to quantify the degree of hair growth (Figure 6C). Hair follicle lengths showed more significant increases in ACA-treated mice (50 and 100μM of ACA) than in control mice (Figure 6D). These results indicated that ACA has good therapeutic activity in the AGA mouse model.

## 3. Discussion

It is well established that type Ⅱ 5*α*-reductase plays an important role in the miniaturization of hair follicles and male hair loss, and thus inhibitors of type Ⅱ 5*α*-reductase including dutasteride and finasteride have been developed for conventional treatment of AGA [16]. However, long-term treatment has various adverse effects such as impotence, decreased libido, erectile dysfunction, testicular pain, and ejaculation disorders. Therefore, consumers suffering from hair loss desire novel target therapy without these side effects.

Several lines of evidence indicate that natural products can be applied to prevent and treat hair loss. Extracts of *Camellia sinensis*, *Panax ginseng*, and *Hibiscus rosa-sinensis* have been reported to stimulate hair growth in animal models [17]. In addition, decursin, a coumarin from *Angelica gigas*, showed a hair-growth-promoting effect in male C57/BL6 mice by decreasing inflammatory cytokines, such as tumor necrosis factor α (TNF-α) and interleukin (IL)-1β, and stimulating anti-inflammatory cytokines including IL-4 and IL-13 [18]. The widely known compounds in *P. ginseng*, ginsenosides Rb1 and Rg3, improved the testosterone-mediated growth suppression of hair matrix keratinocytes in hair follicles [19]. As natural products possess complexity from diverse constituents in the extracts, isolation of active compounds and elucidating activities of each constituent is required to find a novel mode of action in developing a therapy for hair loss.

Generally, oxidative stress is closely associated with hair loss, and many studies have reported experimental data on regulating oxidative stress using natural substances [13]. One well-known natural compound is apocynin, extracted from the roots of *Picrorhiza kurroa* [20,21]. It is known to interfere with the binding of p47phox to gp91phox. However, despite its Nox inhibition activity and antioxidant activity, severe toxicity has been reported, limiting its clinical application. Additionally, compounds such as resveratrol from red wine [22], ginsenosides from ginseng [23], and piceatannol from blueberries [24] have been shown to inhibit Nox activity. However, these compounds primarily exhibit antioxidant effects and indirectly regulate intracellular ROS levels rather than directly modulating Nox activity. Thus, most substances derived from plants have exhibited antioxidant effects and their antioxidant effect on hair loss is very limited. Therefore, our study aimed to discover natural Nox inhibitors and elucidate the mechanism of hair loss by regulating intracellular ROS levels using these compounds. The in silico modeling of ACA with Nox indicates that ACA binds to the cavity where NADPH attaches, directly inhibiting electron transfer to FAD, thereby demonstrating a mechanism that actively suppresses Nox activity (Figure 3). Thus, ACA demonstrated inhibitory effects by binding to the active site of Nox, suggesting that it could ultimately be used as a substance to suppress testosterone-induced hair loss. Additionally, because ACA has a different mechanism of action (MOA) compared to existing drugs like minoxidil or finasteride, it may enhance efficacy when used in combination therapy.

Nox isozymes are one of the significant sources for ROS generation and expression levels of Nox isozymes, and their regulators are associated with various diseases, including diabetic nephropathy, non-alcoholic steatohepatitis, and hair loss [25,26]. In pathological hair loss, Nox isozymes are overexpressed in hair follicle cells, resulting in uncontrolled ROS generation [10,14]. Therefore, uncontrolled Nox activation should be suppressed by treatment with Nox inhibitor as an emerging therapy for hair loss. On the other hand, levels of cellular antioxidants in the body are also associated with hair loss. Activation of the nuclear factor (erythroid-derived 2)-like 2 (Nrf2) pathway can regulate the expression of antioxidant enzymes, including glutathione reductase, peroxiredoxin 1, heme oxygenase-1, and NADH dehydrogenase [27]. Sulforaphane (SFN) from broccoli (*Brassica oleracea* var. *italica*) has been shown to have antioxidant activity through activation of Nrf2 and reduce anagen-to-catagen transition [27,28]. SFN can significantly ameliorate ROS-dependent apoptosis of matrix keratinocytes and reverse ROS-induced reduction in hair matrix proliferation. These results indicate that Nrf2 activators such as SFN might be another therapeutic target for reducing damage caused by excess cellular ROS levels.

**Figure 7:**
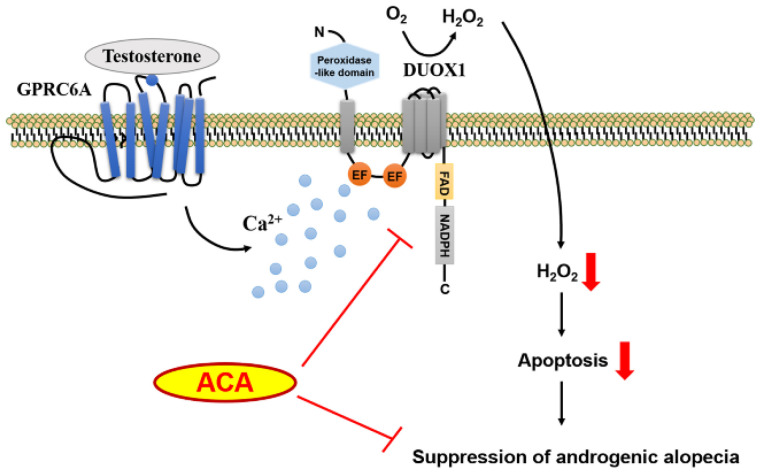
Proposed model for function of ACA in androgenetic alopecia. Binding of testosterone to GPRC6A stimulates downstream signaling networks including intracellular calcium mobilization and Nox activation. Nox-mediated ROS generation mediates apoptosis of keratinocytes, leading to hair loss. ACA suppresses Nox activity and eventually regulates hair loss. The red arrow indicates that each item is decreasing.

In summary, the interaction of testosterone with GPRC6A can stimulate intracellular calcium mobilization, leading to the generation of H_2_O_2_ through Duox1 in skin keratinocytes (Figure 7). Our results show that a natural Nox inhibitor, ACA inhibits such testosterone-induced ROS generation and apoptosis of keratinocytes. In human follicle organ culture, ACA ameliorates testosterone-dependent suppression of hair growth. Topical application of ACA on dorsal skins of testosterone-stimulated C57BL/6 suppressed testosterone-induced hair loss. These collective findings concerning the mode of action and proof-in-concept indicate that ACA is a good therapeutic candidate for male hair loss treatment.

## 4. Materials and Methods

### 4.1. Materials

Thirty-eight pure compounds were isolated from plant materials by one of the authors, Prof. Eun Kyoung Seo, at the Natural Product Chemistry Lab, College of Pharmacy, Ewha Womans University. ACA (**26**) was isolated from *Alpinia galanga* (L.) Will. (Zingiberaceae) by following the previous study [29], and a commercial compound (ab142417) from Abcam (Cambridge, UK) was confirmed to be identical to the isolated compound ACA (**26**). Testosterone (17β-hydroxy-3-oxo-4-androstene; T1500) was purchased from Sigma-Aldrich (Milwaukee, WI, USA) via Sam Eung Industrial Company (Seoul, Republic of Korea). β-Nicotinamide adenine dinucleotide phosphate hydrate (NADPH; N5755) was purchased from Sigma-Aldrich. 2′,7′–Dichlorofluorescin diacetate (DCF-DA; D-399) was purchased from Molecular Probes.

### 4.2. Nox Inhibitor Screening

Nox1, 2, 4, or Duox1 inhibitory activities were determined in a novel assay involving natural compounds. To specifically inhibit Nox isozymes, transgenic *Drosophila* Duox knockdown lines were established that over-expressed human Nox1, 2, 4, or Duox1 [30]. The genotypes used were human Nox1 (UAS-hNOX1/UAS-DUOX-RNAi; Da-GAL4/+), Nox2 (UAS-hNOX2/UAS-DUOX-RNAi; Da-GAL4/+), Nox4 (UAS-hNOX4/UAS-DUOX-RNAi; Da-GAL4/+), and Duox1 (UAS-hDUOX1/UAS-DUOX-RNAi; Da-GAL4/+). Transgenic flies were homogenated with ice-cold PBS containing protease inhibitors and membrane-enriched human Nox1, 2, 4, or Duox1 were harvested. Membranes containing human Nox1, 2, 4, or Duox1 served to monitor ROS production by lucigenin chemiluminescence in the absence or presence of natural compounds [31]. The reaction medium consisted of HEPES-buffered salt solution (145 mM NaCl, 4.8 mM KCl, 1.2 mM MgSO_4_, 1.0 mM KH_2_PO_4_, 1.75 µM CaCl_2_, 0.03 Na_2_EDTA, 5.5 mM glucose, and 10 mM HEPES, pH 7.4), 400 µM lucigenin (10,10-dimethyl-bis-9,9-bisacridinium nitrate) and 500 µM NADPH.

### 4.3. In Silico Model of the Dehydrogenase Domain of hNox5 in Complex with ACA

Using the crystal structure of Nox5 originating from *Cylindrospermum stagnale* (*cs*NOX5) as a template, the hNox5-DH structure was modeled with the Swiss-Model server [32]. The hNox5/FAD complex was modeled by substituting *cs*Nox5 with hNox in the crystal structure of the *cs*Nox5/FAD complex (PDB code: 5O0X). The structure of the ACA was prepared using ChemDraw 18.0 3D software (Perkin Elmer Informatics, Waltham, MA, USA). Molecular docking of ACA against the hNox5-DH was performed using AutoDock Vina software (Forte, S. AutoDock Tools (version 1.5.6 rc2), Molecular Graphics Laboratory, Scripps Research Institute (http://mgltools.scripps.edu, 30 June 2018) [33].

### 4.4. Cell Culture

Ker-CT cells (RL-4048) were purchased from ATCC^®^ (Manassas, VA, USA) and cultured in EpiLife^TM^ medium (MEPI500CA, Gibco, Waltham, MA, USA) supplemented with EDGS (EpiLife^TM^ Defined Growth Supplement; S0125, Gibco) and 1% penicillin-streptomycin solutions (LS202-02, Welgene, Gyeongsan, Republic of Korea).

### 4.5. Measurement of Intracellular ROS by DCF-DA

After cells were starved overnight in a serum-free medium, they were stimulated by the inducer, testosterone, with or without ACA. Cells were washed with Hanks’ balanced salt solution (HBSS) and incubated with HBSS containing 10 µM 2′,7’-DCF-DA for 10 min. The fluorescence was measured using an LSM510 confocal microscope at an excitation wavelength of 488 nm and an emission wavelength of 515–540 nm. Five groups of each type of cell were randomly selected, and the mean relative fluorescence intensity was measured with a Carl Zeiss vision system (LSM510, version 2.3). All experiments were repeated at least three times.

### 4.6. TdT-UDP Nick End Labeling (TUNEL) Assay

Apoptotic cells were detected by the TUNEL technique using an In Situ Cell Death Detection Kit, Fluorescein (11684795910, Roche, Basel, Switzerland). Cells were incubated with testosterone with or without ACA for 24 h. They were fixed with 3.5% paraformaldehyde for 1 h at room temperature and permeabilized with pre-chilled 0.5% triton X-100 in PBS for 10 min at room temperature. These cells were then stained with 4′,6-diamidino-2-phenylindole (DAPI) for 10 min and mounted with mounting solution (M1289, Sigma-Aldrich). Fluorescence was measured by confocal microscopy. Samples were randomly detected over four points, and the percentages of apoptotic cells were determined by counting the numbers of positively stained cells using ImagePro Analyzer 7.0 software.

### 4.7. Function of ACA in Testosterone-Treated Human Hair Follicle Organ Culture

Non-balding scalp specimens were obtained from non-balding occipital scalps of male patients undergoing hair transplantation surgery for AGA at Kyungpook National University Hospital (IRB number KNU-2021-0113). To isolate hair follicles, subcutaneous fat portions were dissected from the epidermis and dermis. Isolated hair follicles were cultured in Williams E media (12551-032, Gibco) supplemented with 2 mM L-glutamine, 100 U/mL streptomycin, and 10 ng/mL hydrocortisone without insulin. All processes were performed following methods reported previously [34], with minor modifications. Hair follicles were incubated with or without testosterone and ACA for 6 days.

### 4.8. ACA Efficacy Test in Testosterone-Treated Dorsal Skin of C57BL/6J Male Mice

To estimate the effects of ACA on AGA, C57BL/6J male mice were anesthetized with isoflurane gas, and their dorsal hair was shaved. Hairs were entirely removed with appropriate amounts of hair removal cream. The next day, scar-less mice were collected and treated with testosterone (200 µg/100 µL) for 3 days. Then, ACA (50 µM and 100 µM) and the same volume of DMSO were diluted with 70% ethanol and administered to each group of mice with testosterone. The treatment was repeated once per day for 12 days. Mice were then sacrificed and their dorsal skins were collected. Tissues were frozen in frozen section media (Tissue-Tek OCT compound, Catalog number: IA018, Sakura Finetek, CA, USA) at −80 °C. Sections were prepared at a thickness of 10–12 µm.

### 4.9. Statistical Analyses

All values are presented as mean ± SD or SEM. Significant differences between groups were determined using a two-tailed Student’s *t*-test. *p*-values less than 0.05 were considered statistically significant.

## Data Availability

The data will be made available on request.

## Abbreviations

The following abbreviations are used in this manuscript:

ACA	1′S-1′-Acetoxychavicol acetate
AGA	Androgenetic alopecia
DP	Dermal papilla
Ker-CT	hTERT/CDK4 immortalized human keratinocytes
Nox	NADPH oxidase
ORS	Outer root sheath
ROS	Reactive oxygen species
TUNEL	TdT-UDP nick end labeling
DCF-DA	2′,7′-dichlorodihydrofluorescein diacetate
